# Protection Against Solar Ultraviolet Radiation in Outdoor Construction Workers: Study Protocol for a Non-randomized Controlled Intervention Study

**DOI:** 10.3389/fpubh.2021.602933

**Published:** 2021-03-04

**Authors:** Anne J. Keurentjes, Sanja Kezic, Thomas Rustemeyer, Carel T. J. Hulshof, Henk F. van der Molen

**Affiliations:** ^1^Amsterdam University Medical Centers, University of Amsterdam, Department of Public and Occupational Health, Amsterdam Public Health Research Institute, Amsterdam, Netherlands; ^2^Department of Dermatology and Allergology, Amsterdam University Medical Centers, Amsterdam, Netherlands

**Keywords:** outdoor workers, solar radiation, intervention, non-melanoma skin cancer, use of sunscreen, occupational disease, stratum corneum, biomarkers

## Abstract

**Introduction:** Non-melanoma skin cancer (NMSC) incidence is increasing, and occupational solar exposure contributes greatly to the overall lifetime ultraviolet radiation (UVR) dose. This is reflected in an excess risk of NMSC showing up to three-fold increase in outdoor workers. Risk of NMSC can be reduced if appropriate measures to reduce UVR-exposure are taken. Regular use of sunscreens showed reduced risk of NMSC. However, sun-safety behavior in outdoor workers is poor. The objective of this study is to investigate the effectiveness of an intervention aiming at increasing sunscreen use by construction workers.

**Methods:** This non-randomized controlled intervention study is comprised of two intervention and two control groups recruited at four different construction sites in the Netherlands. The study population comprises ~200 construction workers, aged 18 years or older, followed during 12 weeks. The intervention consists of providing dispensers with sunscreens (SPF 50+) at construction sites and regular feedback on the application achieved by continuous electronic monitoring. All groups will receive basic information on UV-exposure and skin protection. Stratum corneum (SC) samples will be collected for measurement of biomarkers to assess internal UV-dose. External UV-dose will be assessed by personal UV-sensors worn by the workers during work-shifts in both groups. To detect presence of actinic keratosis (AK) or NMSC, a skin check of body parts exposed to the sun will be performed at the end of the study. The effect of the intervention will be assessed from data on self-reported sunscreen use by means of questionnaires collected on baseline and after 12 weeks of intervention (primary outcome). Levels of SC biomarkers of internal UV-dose, external UV-dose, number of sunburn episodes, and prevalence of NMSC including AK will be assessed as secondary outcomes. The electronically monitored sunscreen consumption will be assessed as process outcome.

**Discussion:** This study is intended to provide evidence of the effectiveness of a technology-driven intervention to increase sunscreen use in outdoor construction workers. Furthermore, it will increase insight in the UV-protective behavior, external and internal UV-exposure, and the prevalence of NMSC, including AK, in construction workers.

**Trial Registration:** The Netherlands Trial Register (NTR): NL8462 Registered on March 19, 2020.

## Introduction

Globally, non-melanoma skin cancers (NMSC) are the most common cancers in fair-skinned populations (1). Solar ultraviolet radiation (UVR) is the main cause of NMSC in fair-skinned people, responsible for ~50–70% of squamous cell carcinoma (SCC) and 50–90% of basal cell carcinoma (BCC) (2, 3). Recently, systematic reviews found that the risk among outdoor workers was raised for SCC and actinic keratosis (AK) by 77%, and for BCC by 43% respectively, compared with the general population (4, 5). Occupational solar exposure contributes greatly to the overall lifetime UV dose. This is reflected in an excess risk of NMSC showing up to three-fold increase in outdoor workers (6). High and increasing incidence rates and frequent recurrence have considerable impact on life and productivity of affected workers. This burden is recognized by the World Health Organization (WHO) and the International Labour Organization (ILO) in a recently published a protocol for a systematic review on the effect of occupational UVR-exposure on NMSC prevalence (7). In six EU countries NMSC has been recognized as an occupational disease (8). NMSC can largely be avoided if appropriate measures to reduce UVR are taken. Several prevention strategies have been developed based on educational programs or use of sunscreens and protective clothing such as long-sleeved shirts and wide-brimmed hats (9). Sunscreen is shown to be an efficient strategy to reduce UVR exposure and its consequences (10, 11). It is a feasible measure to adopt by outdoor workers (12–14), and when used regularly, sunscreens are able to prevent the formation of actinic keratosis and eventually squamous cell carcinoma (10, 11). However, previous research revealed several barriers to using sunscreen, such as the common belief that people with a tanned or dark skin are not at risk for skin cancer and protective measures are not necessary (10, 15), or that sunscreens are expensive (16). Also, generally positive attitudes toward a tanned skin are associated with a decrease in sunscreen use, preventing outdoor workers from taking sun protection seriously (10, 17). Putting on sunscreen is seen as a disturbance and a nuisance, for example it is messy and time-consuming to apply (10, 16, 17). Furthermore, many outdoor workers are male and they may feel it is not masculine to protect themselves from the sun (10, 18), especially around other men (10, 19). Sun-safety behavior among outdoor workers is still poor (10, 20, 21), with examples of 75% of operating engineers seldom or never using sunscreen and 80% of those workers reporting sunburns during the summer (10). However, in another study the majority of outdoor workers did use sunscreen during the summer but they used it incorrectly regarding time, frequency and amount applied (21). Additionally, a recently published meta-analysis showed that the most effective intervention for promoting sunscreen use is providing free sunscreen (22).

Several gaps in the current knowledge are to be filled, these are the prevalence of NMSC including AK, the occupational UV-exposure, and ultimately the effectiveness of an intervention aimed at increasing of sunscreen use in outdoor workers. Well-designed and sufficiently powered studies with adequate adjustment for confounding factors are required to provide more accurate risk estimates for occupational NMSC (23). Data on UV-exposure (external and internal, i.e., the UV-dose that reaches the surface of the skin and the UV-dose absorbed by the skin, respectively), presence of NMSC (including AK), and sunscreen use in outdoor workers in the Netherlands are not yet available.

### Objectives

The objectives of this study are (i) to evaluate an intervention consisting of the facilitation of sunscreen dispensers with continuous electronic monitoring and feedback on the use of sunscreens at worksites, and (ii) to assess occupational UV-exposure and the prevalence of NMSC, including AK, among construction workers.

### Hypothesis

We hypothesize that provision of sunscreen dispensers (facilitation), accompanied by continuous monitoring and intermittent feedback on sunscreen use (awareness and feedback), will significantly increase the use of sunscreen amongst construction workers compared to a control group.

## Methods/Design

### Design and Setting

This is a non-randomized controlled intervention study in construction workers. The duration is 12 weeks, from May to July. The measurements will consist of questionnaires, clinical and biochemical assessments, personal UV-dosimetry, and continuous electronic sunscreen consumption records. When reporting the results of this study we will adhere to the Transparent Reporting of Evaluations with Non-randomized Designs (TREND) statement (24).

A nationwide construction company in the Netherlands will appoint four comparable construction sites suitable for the study. Two sites will serve as the intervention groups and the other two construction sites will serve as the control groups. To minimize potential bias induced by non-randomization, the control groups will be matched to the intervention groups regarding worksites and job tasks, geographical location of the worksites, and time-frame. To avoid contamination bias, the whole construction site will be assigned en masse to the intervention group and there will be no rotation of workers between the four workplaces.

A process evaluation of the intervention will take place in the closing questionnaire to support a future implementation process.

### Participants and Recruitment

The participants in this study are construction workers, engaged in outdoor work activities. The participants are aged 18 years or older, have expressed the willingness to comply with the study protocol, and provided informed consent (inclusion criteria). The construction workers will be recruited by the occupational health service of their company. The construction workers will receive a letter from the investigators stating the purpose of the study, a short version of the study protocol, and a brief description of the expected burden for the participant during the intervention. Information regarding the intervention will be omitted for the participants in the control groups. The participants will be advised to contact the independent physician if they have additional questions regarding health risk associated with the study. The participants will be asked for their consent by the investigator and sign an informed consent form. The participants will have at least 24 h to consider their decision.

### Products Used in the Intervention Groups

The electronic dispensers placed on the construction sites in the intervention groups will be filled with sunscreen Stokoderm® Sun Protect 50 PURE SPF 50 UV skin protection lotion for professional use. This product is a cosmetic product regulated by and complying with Regulation EC no. 1223/2009 (as amended) on Cosmetics Products. The main ingredients are ethylhexyl salicylate, bis-ethylhexyloxyphenol methoxyphenyl triazine, butyl methoxydibenzoylmethane, octocrylene, and homosalate.

### Description of the Study Procedures and Intervention

**Figure 1:**
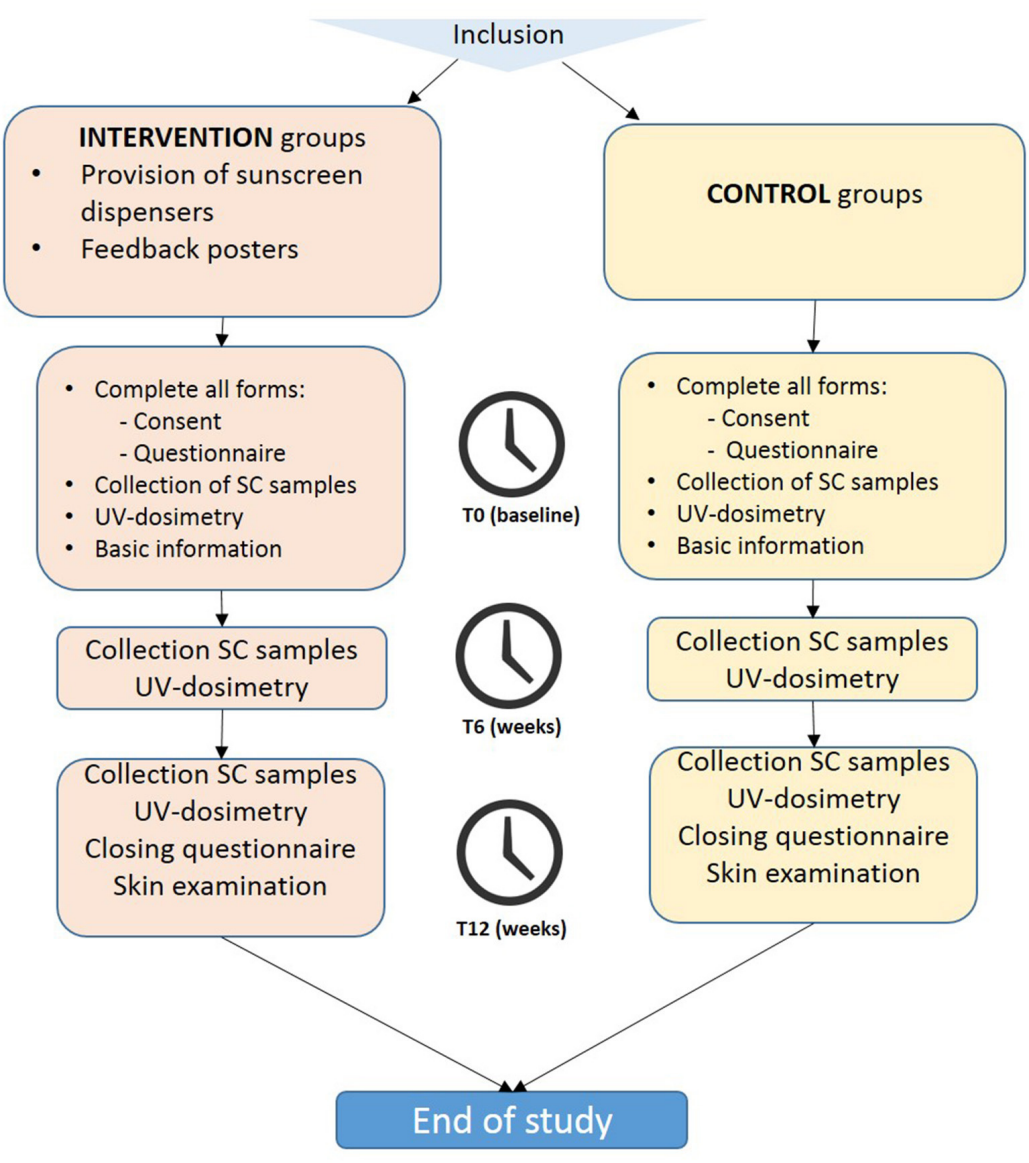
Flowchart of the study design.

The flowchart of the study design is shown in Figure 1. During the recruitment phase, the researchers will visit the construction sites. The construction workers will be informed on the study protocol in both oral and written form by the investigator. Written informed consent will be obtained. The suitability of the construction worker to participate in the study will be checked using the inclusion criteria, as mentioned before (in section Participants and Recruitment). Construction workers fulfilling the criteria will be enrolled in the study.

The researchers will visit the worksites of the intervention and control groups three times (at T = 0, T = 6 weeks, and T = 12 weeks). The participants will be asked to fill in a questionnaire at the start (T = 0) and the end of the study (T = 12 weeks). At T = 0, T = 6 weeks, and T = 12 weeks, stratum corneum (SC) samples will be collected, and measurements of personal UV-exposure by using personal dosimeters will be performed during work shifts for 1 week (Monday to Friday). At the end of the study (T = 12 weeks), the participants will undergo a skin check of the sun-exposed body parts for the presence of AK and NMSC by a trained investigator (physician). The intervention and control groups will receive basic information (i.e., a 15-min Powerpoint presentation) on the nature of the study and sun-safety and UV-protective behavior at the beginning of the study (baseline).

#### Questionnaires

The questionnaire will include questions about age, gender, country of origin, work status as outdoor worker and job characteristics (e.g., job task), number of years in current profession and previous jobs, sun-related risk knowledge, attitudes, barriers for using sunscreen, outside leisure-time spending, and UV-protective behaviors (e.g., use of sunscreen). In the closing questionnaire (T = 12 weeks) an additional question about the number of sunburn episodes during the study period will be included. In the intervention groups questions about satisfaction with the provided sunscreen and their opinion about the effectiveness of the feedback posters will be added (see Intervention Groups: Sunscreen Dispensers and Feedback Posters).

#### UV-Dosimetry

A limited number of participants in all groups (*n* = 10 per each group) will wear a UV-dosimeter during their work shift during 1 week at each time point (T = 0, T = 6 weeks, T = 12 weeks). The selection of outdoor workers who will wear the dosimeters will be performed in way that ensures a maximal variability of job tasks. The Scienterra UV-dosimeter, which will be used in the present study, has proved to be a reliable method to measure external UVB-exposure in outdoor workers (25), and has been used previously to study the influence of human behavior on personal UV-exposure (26). The personal UV-dosimeter will be worn on the left upper arm, which has shown to be a reliable, practical and convenient body site in a previous study (27), and it will not interfere with work tasks.

#### Collection of SC Samples: Procedure of Tape Stripping

During the study, SC samples will be collected at the beginning (T = 0), half-way (T = 6 weeks), and at the end of the study (T = 12 weeks) in both groups. The SC will be collected by using adhesive tape strips with a minimally invasive, non-painful method which is extensively used in experimental studies (28–30). Adhesive tape discs (3.8 cm^2^, D-Squame; CuDerm, Dallas, TX, USA) will be attached to the skin. Each tape is pressed on the skin for 5 sec with standardized force, using a disc pressure applicator (CuDerm). The tape strips will be removed gently with tweezers and stored in a closed vial at −80°C until analysis. The samples will be taken from skin sites exposed to the sun (i.e., forehead), and a less-exposed skin site (i.e., behind the ear).

#### Analysis of the Markers of the Internal UV-Dose

The markers of the internal UV-dose will include the *cis-* and *trans-*isomers of urocanic acid (UCA), and immune markers of different signature such as matrix metalloproteinases (MMP), cytokines, and angiogenesis factors. The isomers of UCA are one of the most studied UVR-related biomarkers (31–34). *Trans*-urocanic acid (tUCA) is a major UVR-absorbing component in the epidermis and it isomerizes to the *cis-*form (cUCA) upon exposure to UVB in a dose-dependent manner until reaching a photo stationary state at ~60–70% of total UCA (35). That makes cUCA a very specific marker as it is not endogenously present but is formed upon exposure to UVR (36). Immunological markers have been proposed to assess the effects of UVR-exposure (37–40), as the adverse effects of UVR might have occurred before visible changes occur (erythema of the skin), and furthermore, immune response in the skin plays an important role in UVR-mediated damage (41). Immunological markers might be in particular useful to assess repeated exposure to UVR (29).

The markers will be extracted from the tape using a buffer, and subsequently analyzed using an appropriate technique. For urocanic acid, HPLC (High Performance Liquid Chromatography) method will be used, and for cytokines the multiplex immuno-assay (MSD–Meso Scale Discovery LLC, Maryland, U.S.A.). For all analyses, standard operating procedures (SOP) will be used. The analysis of the markers will be performed blinded, the samples will be coded untraceable to the participants (the codes will be open after data analysis has been performed).

#### Skin Check

At the end of the study (T = 12 weeks), a skin check of the sun-exposed skin by a trained investigator (physician) will be performed on the participants of all groups. Besides examination for NMSC and AK, following clinical features (42), skin photo type following Fitzpatrick (43) will be recorded. Furthermore, skin photo damage will be assessed by the validated Glogau photo damage classification scale (44).

#### Intervention Groups: Sunscreen Dispensers and Feedback Posters

The intervention groups will be provided with electronic sunscreen dispensers (with monitoring system) installed at the construction site at readily accessible strategic places (canteen/offices etc.). The electronic dispenser, equipped with a Wi-Fi transmitter, continuously records each application event, providing information on the timing and frequency of sunscreen use during the work shift. The system provides robust and easy to interpret web-based reports on sunscreen use per dispenser. Data on use pattern (frequency, total consumption, moments of use) and trends will enable structured feedback on sunscreen use to be given to the construction workers to motivate and improve compliance. Feedback on sunscreen use will be provided using posters placed in proximity of the dispensers, and will be replaced with actual data every 2 weeks. To increase the readability and understanding of the information on the posters, visual aids will be used when possible. Recent systematic reviews found that processing a message in a colorful and illustrative format transmits the message more effectively (45, 46). Also, with the increase of foreign nationals in construction, the use of visual means for conveying health and safety messages is widely popular (45).

### Outcome Measures

#### Primary Outcome

The individual frequency of sunscreen use will be derived from the questionnaires. When asked how often sunscreen is applied on a daily basis in the last month, the answer options are “never, seldom, sometimes, often, always.” Difference in the frequency of sunscreen use between the intervention and control groups will serve as the primary outcome.

#### Secondary Outcomes

Several secondary outcomes will be assessed:

- Internal UV-dose will be determined by measuring the SC levels of UCA isomers and immunological markers measured in the intervention and control groups at T = 0, T = 6 weeks, and T = 12 weeks.
- Levels of external UV-exposure in construction workers, measured using Scienterra UV-dosimeters at T = 0, T = 6 weeks, T = 12 weeks.
- The prevalence of NMSC including AK in construction workers as assessed at T = 12 weeks by a skin check.
- The number of reported episodes of sunburn during the study period as obtained from the closing questionnaire at T = 12 weeks.

#### Process Outcomes

- Pattern of sunscreen use derived from data generated by the electronic monitoring system of the sunscreen dispensers, in the intervention groups only. This will include frequency (averaged for the number of workers), time of use, association with UV-exposure and job task, averaged per person and day.
- Pattern of sunscreen use in relation to the time after placing a poster with feedback concerning UV-index and sunscreen consumption (in the intervention groups only, derived from electronic monitoring).
- Satisfaction with the intervention by the construction workers and employers as assessed by the closing questionnaire. The questions concern satisfaction with the sunscreen (ease of use, ability to perform job task etc.), and satisfaction with the placement of the dispensers (in the intervention groups only).
- Changes in UV-protective behavior regarding sunscreen use. This will be assessed from the questionnaires collected at T = 0 and T = 12 weeks from the questions related to attitude and motivation to use sunscreen.
- Identification of possible barriers to using sunscreens will be assessed from the questionnaires collected at T = 0 and T = 12 weeks. The questions address barriers such as difficulty to implement in the work shift, disturbance of work tasks or negative comments from colleagues when applying sunscreen.
- Knowledge about UV-exposure and UV-protection that will be assessed from the questionnaires at T = 0 and T = 12 weeks. Questions include awareness that applying of sunscreen is important even on cloudy days or on already tanned skin.

### Power Calculation

The study is planned to include 200 participants. The sample size is based on the expectation regarding the change in sunscreen usage. There is no available data on sunscreen use in outdoor workers in the Netherlands, or the barriers for sunscreen use. Therefore, we based our calculations of the sample size on a Canadian study reporting that 25% of the outdoor workers used sunscreen regularly (47). We assumed that 25% of the outdoor workers in the control groups will use sunscreen, and that in the intervention groups we expect this percentage will increase up to 50%. To calculate the sample size, nQuery Advisor software (Statistical Solutions Ltd, Boston, MA, U.S.A.) was used (proportion, two groups, two-sided test). A sample size of 58 workers per group will have 80% power to detect a difference in proportion that equals at least 0.05 significance level. Taking into account possible drop-outs, 100 outdoor workers per group will be recruited.

### Statistical Methods and Data Analysis

There will be no replacement of any individual subjects who withdraw. However, the characteristics (e.g., job task, age) and number of withdrawals will be monitored.

The characteristics of the construction workers (e.g., age) and job tasks will be presented by using descriptive statistics. We will use the mean and standard deviation to describe normally distributed continuous variables and the median and interquartile range to describe non-normally distributed continuous variables. For the self-reported sunscreen usage data (primary outcome), counts and percentages to present categorical variables will be used. The self-reported sunscreen usage data will be dichotomized and analyzed by Chi-squared statistical test to establish whether sunscreen consumption will differ between the intervention and control groups. Two-sided *p*-values of <0.05 will be considered statistically significant and statistical uncertainty will be expressed using two-sided 95% confidence intervals. For the main study parameter, intention-to-treat analysis will be performed.

For the secondary study parameters we will present the levels of biomarkers and the number of sunburn episodes as quantitative, continuous variables. The biomarker levels at T = 6 weeks and T = 12 weeks will be compared with the baseline levels using paired ANOVA test followed by the correction for multiple testing, dependently on the distribution of data. The presence of NMSC including AK will be presented as counts and percentages.

UV-exposure measured by UV-dosimeters will be presented per job task, and as average of the measurements by all workers who worn the dosimeter in the same period. Data will be presented as average dose per day. Furthermore, the UV-exposure pattern during the work shift will be revealed.

The distribution will be tested by using Shapiro-Wilk normality test.

Before data analysis, a detailed data analysis plan will be available.

### Blinding

Due to the study design and the placement of dispensers on the intervention work sites, it is not possible to blind the participants and investigators. The analysis of the SC samples will be performed blinded, the samples will be coded and the unblinding will be performed after all data are analyzed and archived in the laboratory.

## Discussion

The overall purpose of this study is to evaluate the effectiveness of an intervention comprising the facilitation of sunscreen dispensers and regular feedback on sunscreen use in outdoor construction workers. Next, this study will provide insight in UV-exposure, and prevalence of NMSC including AK in construction workers in the Netherlands.

The effectiveness of the intervention will be assessed from self-reported data on sunscreen use (primary outcome), and the changes in the levels of biomarkers of internal UV-dose measured at baseline and after 6 and 12 weeks (secondary outcome). To evaluate the process of the intervention, electronically monitored sunscreen consumption will be used. Furthermore, satisfaction regarding intervention and main barriers for using of sunscreens will be investigated in construction workers.

The intervention is easy and straightforward, and as such the expectation is, that it should be feasible to implement on construction sites. The results of this study will gain insight into the effectiveness of the intervention on UV-protection, and will provide relevant data on the use of sunscreen in outdoor work situations and on the occupational UV-exposure of construction workers.

Recently, a randomized control crossover trial in the United Kingdom (48) which aimed to reduce UV-exposure in the summer, found outdoor workers were exposed to relatively high UV levels in the summer. From the measured UV-dose, approximately a two-fold increase in the risk of being diagnosed with NMSC could be expected if the exposure continued their whole life. The intervention was based on increasing awareness by sending daily messages on the smartphone with recommendations for appropriate measures to reduce UV-exposure. However, this intervention failed to reduce exposure to UV (48). Another study in the United Kingdom found a slight (non-significant) change in sun protective behavior in construction workers after showing them an educational video (49). Our study is focused on reducing internal UV-exposure by using sunscreen. To remove possible barriers such as availability, accessibility, and the costs of sunscreen (10, 21, 22), we provide sunscreen dispensers placed at easily accessible places. Furthermore, we will electronically monitor the amount of sunscreen used, and provide regular feedback on sunscreen use by means of posters. In general, monitoring and feedback are widely used as a strategy to induce behavior change and have been shown to be effective when baseline performance is low, and it is provided more than once (50). Also group monitoring is recognized as being more effective than monitoring systems based on tracking individual actions which do not exploit the stimulating effect of group coherence (50). However, a recent systematic review found that there is very low quality evidence that company-oriented safety interventions reduce injuries among construction workers, and action is needed to increase the adherence of construction workers and employees to protection measures (51).

Strong points of this study are the real-time monitoring of sunscreen use, facilitation of sunscreens, feedback on sunscreen use, and the objective assessment of external and internal UV-dose by using, respectively UV-dosimetry and biomarkers of UV-dose. Also, assessment of the prevalence of NMSC including AK in outdoor workers by a physician will provide evidence on the prevalence of occupational skin cancer in construction workers.

A limitation of this study is the lack of randomization, which was not feasible. However, the intervention and control groups will be matched regarding same sample size, working environments, and job tasks. The risk of contamination bias is limited because the participants work on different and separated work sites, and therefore are not influenced by the other groups. However, we will give basic information on sun-safety and UV-protective behavior at the beginning of the study (baseline) in the control groups also, therefore this might lead to change in sun-protective behavior. Nevertheless, we cannot withhold basic information from the control groups for ethical reasons. The risk of recall bias cannot be entirely avoided because we use questionnaires to measure the primary outcome, however, we counteract by limiting the timeframe through asking questions concerning 1 month in the past. Lastly, it is known that the body location of the UV-dosimeter has an impact on the measured exposure (52). However, we use UV-dosimetry only on one body location (i.e., the upper left arm) because this is practicable for construction workers, and this is the same body location as a large European study (27) used which makes comparison of UV-exposure between our studies and other countries more feasible.

### Study Status

Recruitment for this study had not started at the time of submission.

## Ethics Statement

The study will be conducted in concordance with the principles of the Declaration of Helsinki (2013), and was approved by the Ethics Committee of Academic Medical Center, Amsterdam, the Netherlands (METC 2020_051/NL72818). Participation is voluntary and written informed consent will be obtained.

## Author Contributions

AK was largely involved in the conception, design, and operational management of the study, prepared the first version of the study protocol and manuscript, and will also be responsible for performing the clinical and biochemical assessments during the study. SK has expertise in skin bioengineering methods for the assessment of stratum corneum samples, involved in the conception, design, and supervision of the trial, and drafting the study protocol and the manuscript together with AK. HM brings expertise in occupational health, pragmatic research, translating research findings into policy, involved in the concept, design, and drafting of the study protocol and manuscript together with AK. TR brings in expertise in dermatology, pragmatic research, and critically reviewed the manuscript. CH brings expertise in occupational health, pragmatic research, and critically reviewed the manuscript. All authors read and approved the final manuscript.

## Conflict of Interest

The authors declare that the research was conducted in the absence of any commercial or financial relationships that could be construed as a potential conflict of interest. The handling Editor declared a past co-authorship with one of the authors SK.

## Abbreviations

AK: actinic keratosis
ANOVA: analysis of variance
BCC: basal cell carcinoma
HPLC: high performance liquid chromatography
MSD: mesoscale discovery
NMSC: non-melanoma skin cancer
SC: stratum corneum
SCC: squamous cell carcinoma
SOP: standard operating procedure
SPF: sun protection factor
UCA: urocanic acid
tUCA: trans-urocanic acid
cUCA: cis-urocanic acid
UV: ultraviolet
UVR: ultraviolet radiation.
